# SG-APSIC1089: Environmental screening of SARS CoV-2 to support an outbreak investigation in Sardjito Hospital, Yogyakarta, Indonesia

**DOI:** 10.1017/ash.2023.24

**Published:** 2023-03-16

**Authors:** Khairul Bariyah, Andaru Dahesihdewi, Yunika Puspa Dewi, Raeni Nursanti

**Affiliations:** Sardjito Hospital, Yogyakarta, Indonesia; Sardjito Hospital, Yogyakarta, Indonesia; Sardjito Hospital, Yogyakarta, Indonesia; Sardjito Hospital, Yogyakarta, Indonesia

## Abstract

**Objectives:** Many healthcare workers and patients in intensive care units of Sardjito Hospital, a referral and academic hospital in Yogyakarta, Indonesia, were infected with SARS-CoV-2 in June–August 2021, during the second wave of the COVID-19 pandemic. Much evidence has shown that SARS-CoV-2 persists on hospital environmental surfaces and medical equipment. We investigated the potential sources of virus in our cases, particularly environmental contamination. **Methods:** Environmental screening for SARS-CoV-2 was conducted using RT-PCR of swabs collected from case-related medical equipment and hospital surfaces. We examined the environmental cleaning method in these areas as well. **Results:** We swabbed medical equipment in close contact with patient droplets such as the ventilator, the high-flow nasal cannula, the nebulizer, and suction equipment, as well as some environmental surfaces near the patient, such as the bed rail, air conditioning unit, and portable HEPA-filter outlet. Among 19 samples, genetic material of SARS-CoV-2 was detected only on a sample from a nebulizer. The point of contamination was on the outer body of that nebulizer, which indicated that the contact transmission source might be from patient droplets and/or inadequate cleaning. No more positive results emerged from our screening, indicating that the environmental cleaning was adequate. The IPC team recommended that we no longer use nebulizers for COVID-19 patients and that the cleaning procedure be improved, particularly after the device is used. **Conclusions:** Environmental screening for SARS-CoV-2 can be used to support investigations of inpatient COVID-19 outbreaks in hospitals. Adequate cleaning and care procedures for medical equipment are very important in preventing the transmission of SARS-CoV-2 in the hospital setting.

